# A Framework of AI-Based Approaches to Improving eHealth Literacy and Combating Infodemic

**DOI:** 10.3389/fpubh.2021.755808

**Published:** 2021-11-30

**Authors:** Tianming Liu, Xiang Xiao

**Affiliations:** ^1^Department of Computer Science, University of Georgia, Athens, GA, United States; ^2^Department of Network and New Media, College of Humanities and Arts, Hunan International Economics University, Changsha, China

**Keywords:** AI, eHealth literacy, education, infodemic, public health

## Abstract

The global COVID-19 pandemic has put everyone in an urgent need of accessing and comprehending health information online. Meanwhile, there has been vast amount of information/misinformation/disinformation generated over the Internet, particularly social media platforms, resulting in an infodemic. This public health crisis of COVID-19 pandemic has put each individual and the entire society in a test: what is the level of eHealth literacy is needed to seek accurate health information from online resources and to combat infodemic during a pandemic? This article aims to summarize the significances and challenges of improving eHealth literacy in both communicable (e.g., COVID-19) and non-communicable diseases [e.g., cancer, Alzheimer's disease, and cardiovascular diseases (CVDs)]. Also, this article will make our recommendations of a general framework of AI-based approaches to improving eHealth literacy and combating infodemic, including AI-augmented lifelong learning, AI-assisted translation, simplification, and summarization, and AI-based content filtering. This general framework of AI-based approaches to improving eHealth literacy and combating infodemic has the general advantage of matching the right online health information to the right people.

## Introduction

Since its conceptualization in Norman et al. (1) in 2006, eHealth literacy, which represents an individual person's capability of finding, understanding, and using relevant health information from online resources, has emerged as an important research and education topic for a variety of related fields, including biomedical informatics, public health, social media, health communication, artificial intelligence (AI), and education, among others. Actually, looking for online health information has gradually become a popular web activity in recent years, akin to other daily web behaviors such as checking email and using search engines (2), and this trend has been dramatically accelerated during the global public health crisis of COVID-19 pandemic (3–6). On one hand, during COVID-19 pandemic, nearly everybody in the whole society needs to access and comprehend online health information than ever before, in order to get prepared for COVID risk mitigation and/or for healthcare management. On the other hand, huge amount of health information, misinformation, and disinformation about COVID-19 has been generated and flowing on the Internet, particularly on social media channels such as Twitter, YouTube, TikTok, Facebook, and others (3–6). As a result, how to differentiate health information/misinformation/disinformation for an average person who might have different levels of eHealth literacy becomes a major social issue for both individual healthcare and public health.

On February 15, 2020, the WHO formally raised the concern that the COVID-19 epidemic was accompanied by an infodemic (7), which is defined as an overabundance of information—some accurate and some not—that occurs during an epidemic (8). Actually, the term infodemic or infodemiology was originally coined in 2002 (9) and was recently deliberated in Eysenbach (5). During the COVID-19 pandemic, the infodemic issue has been intensified to a global scale of the emergency, e.g., many false and misleading claims or incorrect recommendations about prevention or public behavior could be detrimental to global public health. The infodemic problem could be even more challenging to manage when health information is mixed with political narratives and online commentary that is not supported by verified facts and evidence. In response, the WHO invited policy makers, public health professionals, researchers, students, representatives of the media, social media platforms, private sector organizations, and other concerned stakeholders to technical consultation meetings, and summarized a set of 50 proposed actions and revealed six policy implications to manage infodemic in health emergencies (6). It is possible that these actions and implications will improve the quality and accuracy of health information during health emergencies, however, it is unlikely that health misinformation or disinformation will be eliminated.

From an individual's eHealth literacy perspective, it is equally important to accurately comprehend correct health information and to disregard health misinformation/disinformation. However, recent research studies have revealed concerns on the eHealth literacy in various populations and called for major efforts in improving eHealth literacy. It was stated in Paakkari and Okan (10) that the COVID-19 pandemic and infodemic have revealed and highlighted that the whole society has serious problems on eHealth literacy, which has elevated to a public health problem globally. In addition to the import roles of eHealth literacy during COVID-19 pandemic, eHealth literacy is also seen as a crucial tool for the prevention and management of non-communicable diseases such as cancer, Alzheimer's disease, and cardiovascular diseases (CVDs), as already pointed in literature studies (11–13). Importantly, despite their non-communicable nature, prominent diseases like cancer, Alzheimer's disease, and CVDs have impacted large populations and become major public health issues. Therefore, in this article, we would like to summarize the significances and challenges of improving eHealth literacy in both communicable (e.g., COVID-19) and non-communicable diseases (e.g., cancer, Alzheimer's disease, and CVDs) (Section Improving eHealth Literacy: Significance and Challenges), and to make our recommendations of a general framework of AI-based approaches to improving eHealth literacy and combating infodemic, including AI-augmented lifelong learning, AI-assisted language translation, simplification, and summarization, and AI-based content filtering (Section Recommendations). We would like to argue that using AI-based approaches to improving eHealth literacy is essential for alleviating COVID-19 dis/misinformation.

## Improving EHealth Literacy: Significance and Challenges

The eHealth literacy has a relatively short, but active research history^1^, (14, 15). The classic Lily model of eHealth literacy is composed of six core literacies including traditional literacy, media literacy, information literacy, computer literacy, scientific literacy, and health literacy^1^, (14, 15). Typically, the first three literacies are considered as analytic type and the latter three are context-specific^1^. In recent years, it was suggested that the increasing use of social networking media can supplement eHealth literacy (16). During the COVID-19 pandemic, it is apparent that social networking platforms such as Twitter, YouTube, TikTok, Facebook, and others played significant roles in providing and exchanging health information (3–6). In a recently proposed wedding cake model of health information (6), social media is depicted as the largest and last segment of the wedding cake, and it represents the vast amount of nearly unfiltered and uncontrolled information generated or amplified by the public. It was suggested that the main risk of health misinformation/disinformation is not in the science layer of the wedding cake model, but in the translation of science information into actionable recommendations and conveying conclusions for different audiences and stakeholders in other layers such as healthcare practice, news media, and social media (6). Despite many efforts in accurate knowledge translation, knowledge refinement, filtering, and fact-checking, the end users are exposed to health information from any level and in any refinement stage, thus making eHealth literacy an essential skill for everyone in a networked world. This is true for both communicable and non-communicable diseases, thus motivating us to conduct a comprehensive review for both scenarios, with an emphasis on cancer, Alzheimer's disease, CVD, and COVID-19 in this article.

### Significance of EHealth Literacy in Cancer, Alzheimer's, and Cardiovascular Healthcare

Cancer healthcare is a very complex and demanding task that warrants close collaboration between cancer healthcare professionals and patients and their family members. In particular, the levels of eHealth literacy of patients and their family members play major roles in cancer diagnosis, treatment, and follow-up. There have been a vastly amount of research studies on the importance of eHealth literacy in cancer healthcare, as reviewed in a recent article (17). As a result of these studies, a major conclusion is that there are positive associations between eHealth literacy and clinical cancer related outcomes, that is, higher levels of eHealth literacy are associated with a greater possibility of favorable clinical outcomes. On the contrary, inadequate eHealth literacy is associated with lower uptake of screening and preventative behaviors (18, 19), longer lag time in symptom identification and medical help seeking (20, 21), less knowledge of cancer and its prevention and treatment (22, 23), impairments in risk perception (24, 25), greater unmet informational needs (26), less information seeking behaviors (27, 28), lower perceived quality of life (29, 30), less compliance with post-screening or post-treatment follow-up (31, 32), and lower perceived quality and involvement in patient-provider communications (33, 34). Implications of these associations are significant both for patients, their families and the healthcare system in general (17). Therefore, it is apparent that improving eHealth literacy is critically important for better cancer healthcare.

With the increased longevity and improved health at older ages worldwide, healthcare of the elder population and dementia, particularly Alzheimer's disease, presents significant challenges. It was pointed out in Efthymiou et al. (35) that 80% of people with chronic diseases, such as Alzheimer's disease, are cared for at home by a family member, friend, or relative, and thus the eHealth literacies of those home caregivers play a key role in overall healthcare. In the dementia healthcare field, the eHealth literacy was more specifically related to dementia literacy (12), and it is widely recognized that dementia literacy is generally inadequate among patients, older adults, and even health workers. It was argued that caregivers play a crucial role as a health advocate for people with dementia in handling health information and making a shared medical decision (12). In the literature, to measure dementia literacy, a variety of scales were designed. For instance, Carpenter et al. reviewed a pool of items from previous scales designed to assess knowledge about Alzheimer's, dementia, or memory loss and developed the 30-item Alzheimer's Disease Knowledge Scale (ADKS) (36). Annear et al. (37, 38) developed the Dementia Knowledge Assessment Scale (DKAS), which includes four domains of causes and characteristics, communication and behavior, care considerations, and risks and health promotion, and validated the 25-item scale in a cohort of international respondents. Annear et al. reported dementia knowledge deficiencies across different domains which were also identified among healthcare providers (39). Overall, improving eHealth literacy and dementia literacy is much needed for better dementia and Alzheimer's disease healthcare.

Patients with CVD are required to self-manage many aspects of their conditions, which requires eHealth literacy for various tasks. Richtering et al. (40) examined the eHealth literacy predictors of in a population with moderate-to-high cardiovascular risks and suggested the importance of evaluating patients' familiarity with the Internet and the importance of specifically assessing eHealth literacy in conjunction with a health literacy assessment. The COVID-19 pandemic has accelerated the importance of eHealth literacy, as the pandemic required quarantine and isolation and thus face-to-face visits in both primary and secondary cardiovascular care have been hugely reduced. During COVID-19 pandemic, it was learnt that CVD and CVD risk factors are common among hospitalized patients with COVID-19 (41), and thus it is more desirable to embrace eHealth resources that connect cardiovascular care through the internet and related technologies. Also, CVD patients and/or family caregivers need to take active roles and gather data of signs and symptoms (self-care monitoring) and to respond to changes if needed (self-care management). During the pandemic, patient living with CVD are very frustrated and they had so many questions about CVD and COVID-19 and about what they should or should not do (42). Therefore, eHealth literacy is an essential skill for such CVD self-care monitoring and self-care management.

In short, due to complexity, heterogeneity, uncertainty, and chronic nature of diseases like cancer, Alzheimer's, and CVDs, patients and their caregivers need considerable level of eHealth literacy to seek, find, understand, and appraise health information from electronic sources and apply the gained knowledge to addressing or solving specific health problems.

### Significance of EHealth Literacy in COVID-19 Pandemic

For instances, Hong et al. (43) examined the level of eHealth literacy and infection preventive behaviors related to COVID-19 among undergraduate students majoring in healthcare, and they reported that the overall eHealth literacy measures were related to infection-preventive behaviors. The findings in Hong et al. (43) highlighted the necessity of education for improving the eHealth literacy of undergraduate students majoring in healthcare to strengthen infection-preventive behaviors and protect patients from infectious diseases. Dib et al. (44) discussed online false information about COVID-19 vaccine and the vaccine hesitancy, and they advocated that eHealth literacy should be viewed as fundamental skills that have the potential to empower citizens to better recognize online mis/disinformation and make informed health decisions, such as whether to take COVID-19 vaccination or not. An et al. (45) reported that there is a clear and consistent association between higher coronavirus-related eHealth literacy and greater knowledge, lower conspiracy beliefs, and greater engagement in protective behaviors. Paakkari and Okan (10) argued that the COVID-19 infodemic has revealed and highlighted that poor eHealth literacy is an underestimated public health problem globally. For instance, nearly half of adults in Europe reported having problems with health literacy and not having relevant competencies to take care of their health and that of others (10). The reality is that the COVID-19 pandemic has called for people to acquire/apply health information quickly and adapt their behaviors at a fast pace, which significantly increased the demand of eHealth literacy of the public. Therefore, the significance of eHealth literacy during COVID-19 pandemic is further amplified and highlighted.

Notably, there are differences in the challenges and significances of eHealth literacy in communicable (e.g., COVID-19) and non-communicable (e.g., cancer, Alzheimer's, and CVDs) diseases. For instance, the levels of urgence in seeking healthcare-related information and patients' demographic characteristics and social determinants could be different in communicable and non-communicable diseases. Thus, our recommendations in the following section will consider such differences accordingly.

## Recommendations

In general, eHealth literacy means very differently to individuals with diverse backgrounds in traditional literacy, media literacy, information literacy, computer literacy, scientific literacy, and health literacy. Therefore, a key consideration when trying to improve eHealth literacy is to tailor general approaches to individual groups and scenarios. Here, we recommend a general framework of AI-based approaches originated from the methodologies in AI field, and these AI-based approaches have the general advantage of matching the right online health information to the right people.

### AI-Augmented Lifelong Cyberlearning

An individual person's eHealth literacy learning is a complex, lifelong process, which entails applying scientific insights about how people learn, leveraging emerging technologies such as social media and mobile devices, designing and using transformative learning activities, engaging teachers and other practitioners, and emphasizing continuous improvement. All of these components are integral parts of the cyberlearning, which is recently used by the NSF cyberlearning program^2^. In our view, cyberlearning approaches that integrate AI with learning theories to create multimodal mashups will be promising to improve individuals' eHealth literacy. Actually, the concept of mashup for learning was proposed years ago (46), which is a combination of two or more data sources that have been integrated into one source. For instance, mashups could typically consist of graphics, texts, audio clips, and video that have been sourced from various media such as blogs, wikis, YouTube, Google Maps, etc., into a new product for education and learning purpose. In an early effort, Bentley et al. (47) built the Health Mashups system to identify connections that are significant over time between weight, sleep, step count, calendar data, location, weather, pain, food intake, and mood. The authors found that their participant users were able to build an awareness of certain contextual well-being patterns in their lives and focused their change efforts based on this new awareness. Bentley et al. (47) also showed how these effects were very personally unique, with different participants having different sets of significant observations, suggested the importance of building personalized health mashups for individuals. In this context, AI algorithms and methods will play key roles in personal profiling of each individual and in construction of such “ambitious mashups”^3^.

Notably, mashup technologies have been advanced significantly in recent years because of the maturity of the service-oriented computing paradigm and service-oriented software development (48, 49). For instance, by integrating existing Web services that are widely available online, software developers can more easily and efficiently build their mashups, i.e., Web applications that can provide certain functionalities by composing one or more services. More recently, Ma et al. (48) developed a deep learning based interactive service composition framework which aimed to capture the interactions among the target mashup, selected services, and the next service to recommend. In particular, an attention mechanism was employed in this deep learning AI system to weigh selected services when recommending the next service. Therefore, this new AI-augmented mashup construction system can recommend suitable follow-up component services to develop new mashups, thus facilitating dynamic requirements and behaviors (e.g., instant service selection). Given that mashup technologies have wide applicability in the education context^4^, we would like to advocate that mashup approaches, particularly AI-augmented mashup construction systems, should be widely available for dynamic and personalized construction of eHealth literacy education mashups. This powerful technology provides great flexibility and sustainability for complex, diverse, and dynamic needs of health literacy education for different populations with diverse backgrounds. As the US Department of Health and Human Services launched the national action plan to improve health literacy^5^, effort should be made to build up the widely accessible infrastructure of AI-augmented lifelong cyberlearning for eHealth literacy education.

Also, AI-enabled intelligent tutoring systems (ITS) of health information could be considered to support and guide individuals as they learn relevant health information and knowledge from online resources. As reviewed in Mousavinasab et al. (50), uses of ITSs as an adaptive learning tool are increasing significantly across different educational fields, including eHealth literacy education, and adaptive and personalized learning can be achieved based on the learner's knowledge and performance. However, it should be pointed out that ITSs are less capable and effective than human tutors in the areas of dialogue and feedback. For instance, human tutors can interpret the affective or emotional states of the student, and then potentially adapt instruction in response to these perceptions. In recent years, deep learning and AI have been integrated into the development and application of ITS for health education purposes. For instance, Furlan et al. (51) developed a virtual patient simulators (VPS) system that allowed students to gather clinical information from the patient's medical history, physical exam, and investigations and allowed them to formulate a differential diagnosis by using natural language. The VPS is also an ITS that provided real-time step-by-step feedback to the student and suggests specific topics the student has to review to fill in potential knowledge gaps. By combining ITS and natural language processing (NLP) AI technologies, the VPS system can provide medical students with a learning tool for training them in diagnostic reasoning. This type of technology is particularly useful in a setting where students have restricted access to real clinical environment during the COVID-19 pandemic. We believe this methodology can be of great value and potential for improving eHealth literacy and combating infordemic for the general public, e.g., those who are seeking health information for self-care monitoring and self-care management during COVID-19 pandemic. Apparently, this type of AI-augmented ITS system should be tailored based on the stratification of various demographic, clinical, and environmental factors such as age, health condition, and living environment.

Overall, AI-augmented lifelong cyberlearning diagrams and approaches have great potential to transform current landscape of eHealth literacy education and significantly advance the eHealth literacy of the general public, including both healthcare professionals and average individuals. It is without doubt that significant efforts are needed from multidisciplinary domains, including governments, healthcare professionals, educators, computer and information systems, and AI experts, to move this field forward collaboratively and synergistically. It is equally important that individual citizens recognize and appreciate the importance of eHealth literacy for both personal life and public health, and accept the idea that eHealth literacy is a fundamental skill, like writing and reading, as advocated in literature articles (44). In this way, individuals will feel more motivated and incentivized to improve their eHealth literacy and embrace new tools like AI-augmented lifelong cyberlearning tools mentioned above. Also, this AI-augmented lifelong cyberlearning approach might be particularly suitable for non-communicable diseases, e.g., cancer, Alzheimer's, and CVDs.

### AI-Assisted Language Translation, Simplification, and Summarization

In governments' guidelines and checklists regarding online eHealth literacy education materials^5^, it is recommended that those materials should be delivered in ways that are easy to understand and that improve health, longevity, and quality of life, and should be accessible and available to all people who have the right to health information that helps them make informed decisions. However, it is technically challenging that eHealth literacy education materials, e.g., reading materials, are understandable to different populations with very different backgrounds, educations, and language proficiencies. Fortunately, the rapid advancements in NLP AI techniques, particularly machine translation methods, have provided great promise to translate and adopt eHealth literacy materials for diverse populations. Dew et al. (52) conducted a systematic review of literature articles covering 2006–2016 to investigate the degree to which machine translation was being developed for use for health settings and to synthesize evidence concerning where machine translation might be most appropriately used to facilitate communication and provide translated health materials. It was reported that machine translation was developed primarily through pilot studies to improve multilingual communication in health settings and to increase access to health resources for a variety of languages (52, 53).

In the NLP AI field, another important relevant technology is text simplification, which aims to rewrite an input text so that the output is more readable and understandable (54). Simplified text can reduce complexity for NLP and can provide reading aids for people with limited language skills or language impairments such as dyslexia, autism, and aphasia. Natural language processing researchers have developed three major types of classic sentence simplification operations: splitting, deletion, and paraphrasing (55). Technically speaking, text simplification is closely related to other NLP AI techniques, such as machine translation, monolingual text-to-text generation, text summarization, and paraphrase generation (56). Due to the significant advancements of NLP AI in the past few years, particularly deep learning, text simplification methods have accordingly been shifted from traditional, statistical-based methods toward deep learning AI techniques, such as Recurrent Neural Networks (RNNs) (56), Long Short-Term Memory (LSTM) networks (56), and transformers (57). Zhao et al. (57) proposed a novel model based on a multi-layer and multi-head attention architecture for text simplification that covers a wide range of real-world simplification rules. The authors reported that the integrated transformer model outperformed multiple state-of-the-art baseline models for sentence simplification and provided more accurate simplification rules.

In a recent work, Van et al. (58) pointed out that fully automated text simplification might not be appropriate for healthcare information since it is critical that the important information should be guaranteed to be preserved fully during the simplification process. For instance, the Shardlow et al. (59) reported that fully automated text simplification approaches omitted 30% of critical information when simplifying clinical texts. Accordingly, Van et al. (58) developed a new parallel medical data set consisting of aligned English Wikipedia with Simple English Wikipedia sentences and examined the application of pretrained NLP models, such as the popular BERT (60), RoBERTa (61), XLNet (62), and GPT-2 (63)^6^, on this dataset. Also, Van et al. (58) proposed a new autocomplete application for those popular NLP models for medical text simplification. It was shown that such autocomplete models can assist users in simplifying text with improved efficiency and higher quality results in the domains of healthcare and medicine. Therefore, it is encouraging that integrating cutting-edge NLP AI approaches for healthcare text simplification could offer a transformative framework for eHealth literacy education among diverse populations with different reading and understanding capabilities.

As pointed out in the wedding cake model of health information (5), knowledge translation processes translated the information from one audience to another, which maybe are the main mechanisms where information becomes misinformation, given that the interpretation of health information is subject to influence factors such as politics, commercial interests, selective reporting, and misunderstandings. These knowledge translations could happen across all four layers of the wedding cake model (5), for example, across scientific papers, news media, and social media. Here, we would like to propose to employ cutting-edge NLP AI approaches to summarize and simplify scientific papers to messages that can be directly understood by average individuals, so that undesirable influence factors could be avoided during manual translations mentioned above. Technically, there are two major categories of NLP AI approaches for scientific paper summarization: abstract generation-based approaches and citation-based approaches (64). Abstract generation-based approaches aim to automatically generate an abstract of a research paper (65–67), while citation-based approaches target generation of summaries based on a set of citing sentences in other scientific papers pointing to that paper (68–70). Recently, Cai et al. (71) proposed a linguistically enriched SciBERT-based summarization model (named COVIDSum) for COVID-19 scientific papers. In this work, the authors first extracted salient sentences from source papers using heuristic strategies and constructed word co-occurrence graphs based on the selected sentences to capture the linguistic features of the sentences. Then, a SciBERT-based sequence encoder and a Graph Attention Network (GAT) based graph encoder were both adopted to encode sentences and word co-occurrence graphs, respectively. Finally, the above two encodings were fused by using highway networks to incorporate linguistic knowledge into the contextual embeddings of scientific COVID-19 papers, and an abstractive summary of each scientific paper was thus generated. Cai et al. (71) evaluated their NLP AI models on the publicly available COVID-19 open research dataset (CORD-19) (72) [a total of 133,206 scientific papers used in Cai et al. (71)] and reported promising results using well-established evaluation metrics. In general, the proposed COVIDSum in Cai et al. (71) offered a new approach to summarizing health and medical scientific papers for the reading and understanding by general audience.

In a recent study, Bala et al. (73) showed that NLP AI software tool can be used to translate medical notes into plain-language notes that can be more easily perceived by patients, shedding new insights and promise into using NLP AI approaches to converting professional health sentences/messages for eHealth literacy education among average individuals, given that manual translations and communications of health message/information between healthcare professionals and average patients are very time-consuming, costly, and challenging (74). In general, NLP AI models have demonstrated great promises in translating, summarizing, and simplifying health materials for wider access and reading by the general public (52, 57, 71, 73). These AI-assisted capabilities could significantly shorten the chains of translating knowledge and information from scientific papers to public health communications, to social media, and to the general audience, and could significantly reduce the risks of political biases, commercial interests, selective reporting, and misunderstandings. Also, these NLP AI-based approaches are applicable for both communicable and non-communicable diseases. Certainly, the possible biases of NLP AI models need to be sufficiently addressed (75) first before they are adopted and applied in the eHealth literacy education domain.

### AI-Based Content Filtering

Improving eHealth literacy for the whole society, e.g., via the approaches discussed in Sections AI-Augmented Lifelong Cyberlearning and AI-assisted Language Translation, Simplification, and Summarization, is an effective way to increase the efficiency of our healthcare systems, to reduce healthcare disparity, and to combat infodemic. Meanwhile, exploration of quicker approaches to combating infodemic, e.g., the one during the COVID-19 pandemic (6, 7), is urgent. In the literature, AI-based detection of fake news on social media has been extensively studied (76–80). For instance, Nasir et al. (79) proposed a hybrid deep learning model that combined convolutional and recurrent neural networks for fake news classification. The authors evaluated the deep learning AI model on two fake news datasets and reported promising results. Kaliyar et al. (80) proposed a BERT-based (Bidirectional Encoder Representations from Transformers) deep learning approach (FakeBERT) by combining different parallel blocks of the single-layer deep Convolutional Neural Network (CNN) with different kernel sizes and filters with the BERT. The authors argued that such a combination is useful to handle ambiguity, which is the greatest challenge to natural language understanding. The authors' experiments demonstrated that their proposed FakeBERT model outperformed the existing models with a promising accuracy (80). Kolluri et al. (81) presented the CoVerifi system, which combined machine learning techniques and human feedback to assess the credibility of COVID-19 related news on social media. By allowing users' vote feedback on news, the CoVerifi system allowed the release of labeled data as open source and enabled further research to prevent the spread of COVID-19 misinformation. The authors also discussed the deployment of CoVerifi at scale for combating COVID-19 infodemic, given that the explosion of misinformation, disinformation, and hate news related to COVID-19 infodemic on social media has left fact checkers overburdened (81). If these deep learning AI based fake news detection systems are deployed by those popular social network platforms and end users, they can play a major role in filtering misinformation and disinformation, thus substantially reducing the risk of infodemic.

Recently, the topic of detecting fake news about COVID-19 on the Web has also received intense interest, partly due to the fact that many people still prefer to use search engines to find needed information on the Web (82). During the COVID-19 pandemic, people have many questions about the origin of the disease, transmission patterns, prevention measures, treatment options, and cure possibilities, and information sources related to these questions are distributed across the whole Web. Despite that popular search engines such as Google and Bing already applied various mechanisms to filter and rank Web pages and documents before they present the results to end users, there are still many Web misinformation and disinformation sources (82). Mazzeo et al. (82) proposed a new direction for Web fake news classification by the integration of the most commonly used features in fake news detection and features that play an important role in malicious URL detection. The authors argued that this feature engineering aimed to feed the original data and provided new and meaningful feature representations to improve AI algorithms for classification. Mazzeo et al. (82) also applied re-sampling techniques, such as under-sampling and over-sampling, to balance datasets, and compared different AI algorithms based on their performances. In the future, it will be important to construct benchmark datasets related COVID-19 news and fake news, and systematically evaluate different AI approaches such as deep learning models and traditional machine learning models (83). This line of work is particularly important to improve eHealth literacy and combat infodemic during the COVID-19 pandemic.

In addition to the abovementioned advancements in information filtering technology on social networks and Web, it might be still important to have a mechanism to certify whether a health information source is reliable or not. For instance, the Health On the Net (HON) project^7^, ^8^ offers the service of certifying whether a website is reliable and useful in terms of providing medical information on the Internet. Health On the Net Foundation also issued a code of conduct (HONcode) for medical and health websites, and those principles include: authority (information and advice given only by medical professionals with credentials of author/s, or a clear statement if this is not the case), complementarity (information and help are to support, not replace, patient-healthcare professional relationships which is the desired means of contact), confidentiality (how the site treats personal and non-personal information of readers), attribution (references to source of information (URL if available) and when it was last updated), justifiability (any treatment, product or service must be supported by balanced, well-referenced scientific information), transparency of authorship (contact information, preferably including email addresses, of authors should be available), transparency of sponsorship (sources of funding for the site), and honesty in advertising and editorial policy (details about advertising on the site and clear distinction between advertised and editorial material)^8^. Currently, there are over 8,000 HONcode certified websites, including the following popular websites of WebMD, Everyday Health, Drugs.com, and Healthline.

Apparently, other than those 8,000+ NONcode certified websites, there are many more Websites and information sources that provide health information to the general public. From a technical perspective, it is a daunting task to monitor, evaluate, and filter the reliability and usefulness of those health information sources. Again, it will be a fundamental task to improve the general public's eHealth literacy so that each individual will be able to assess the level of reliability and usefulness of such health information sources, and AI-assisted language translation, simplification, and summarization and AI-based content filter can give them a hand whenever needed. In addition to AI-based content filtering, building a transparent, consistent, and trustworthy AI ecosystem is critical and should be considered. In short, our overall recommendations for improving eHealth literacy and combating infodemic include a combination of these three directions of efforts.

## Discussion and Conclusion

Our recommendations made in Section Recommendations are from a technical perspective of AI approaches. Here, we will discuss social and ethical issues related to eHealth literacy and infodemic, including the possibility of integrating eHealth literacy education into the curriculum at all levels, a whole-society approach to eHealth literacy, and healthcare disparities. Finally, we will make concluding remarks on these topics in general.

### Integrate EHealth Literacy Education Into Curriculum

Existing literature has already argued that eHealth literacy is a fundamental skill for any individual, which should be trained and educated across the life span, including K-12, college, and the entire adulthood. For example, the CDC has its website for health literacy education for childcare, early childhood, K-12, and universities^9^. Meanwhile, computer and digital literacy education has also been integrated into K-12 and college curriculum across the United States^10^. It should be beneficial to strengthen health literacy education, digital literacy education, and Internet access, and integrate them together in some way so that eHealth literacy can be simultaneously improved during the entire school trajectory. However, it is non-trivial to develop eHealth literacy education materials and curriculum for K-12 and university students, and significant joint effort by educators and healthcare professionals is warranted in the future. It should be emphasized that eHealth literacy education is a lifelong self-learning process that should be continuously maintained and improved, e.g., by the AI-augmented lifelong cyberlearning tools mentioned in Section AI-Augmented Lifelong Cyberlearning. When an individual or his/her family member meets sudden healthcare or disease situation (e.g., diagnosed with cancer, CVD, or Alzheimer's disease, as discussed in Section Significance of eHealth Literacy in Cancer, Alzheimer's, and Cardiovascular Healthcare), the need of eHealth literacy will jump dramatically during a short period of time, which may drag people into a desperate mode. It would be more advantageous to gain sufficient eHealth literary during school education and by continuous lifelong self-learning.

### A Whole-Society Approach to Improving EHealth Literacy and Combating Infodemic

Despite the effort by the national action plan to improve health literacy^5^, approximately one-third of adults in the United States have limited health literacy (11). Therefore, in addition to the efforts and approaches discussed in Section Recommendations, a whole-society approach is much needed to improve eHealth literacy and combat infodemic. Here, we will use the discipline of healthcare communication as one example to illustrate how this discipline can contribute. Recently, the Policy and Practice subcommittee of the International Association for Communication in Healthcare (pEACH) have identified a number of health communication areas that should be considered for improvements during the COVID-19 pandemic (84). First, how to handle uncertainty in COVID-19 communication is a major challenge due to the novel and rapidly evolving nature of the disease and situation. In general, the general public's concept of risk is likely to be poor, and this contributes to a sense of uncertainty and confusion. Therefore, during COVID-19 health information communication, it is important to emphasize the importance of: (1) honesty during times of uncertainty, (2) being transparent with the public about what is and is not known and the rapid development of new knowledge, (3) the use of clear and consistent language, (4) where possible, by consistent spokespeople who can demonstrate honesty, and (5) confidence in making decisions while demonstrating empathy and concern (84, 85). Second, during COVID-19 pandemic, the key messages about wearing masks, washing hands, maintaining social distances, and vaccination are essential to help combat the pandemic. Unfortunately, the success of health communication during COVID-19 is severely challenged by the infodemic. In the future, much research and policy work are needed to include an evidence-based typology of what misinformation and disinformation is during health information communication. Risk communication from authorities to the general public is vital for the latter to be informed and to be able to act in ways that promote their safety and health. Also, risk communication campaigns should include multidisciplinary communication practices, early consultation, and continuous engagement with stakeholders from linguistically and culturally diverse communities, and built-in evaluation strategies (84). Given the fast development pace of the disease such as new virus variants and emerging new knowledge, it is important for the whole society to synchronize quickly, including AI-assisted language summarization or cyberlearning, policy development, and communication campaigns. Overall, engagement of the whole society in healthcare communication is critical, which partly reflects the whole-society approach to improving eHealth literacy and combating infodemic, as we advocated.

### Healthcare Disparity

Healthcare disparity has been recognized as a major concern in current healthcare systems around the world, and disparity in COVID-19 pandemic has shown to be significant (45). Literature studies have suggested that eHealth literacy could be a significant factor in healthcare disparities (74). In the future, it will be invaluable to develop eHealth literacy indicators or profiles to identify at-risk populations for targeting tailored health communications and self-management support interventions. These eHealth literacy indicators or profiles can also help healthcare professionals to improve in individual-level care of the patients. For instance, Schillinger et al. (74) analyzed secure text messages sent by diabetes patients to physicians within an integrated system's electronic portal and then used NLP AI methods to generate five unique literacy profiles by employing various sets of linguistic indices. These quantified literacy profiles could be useful for reducing healthcare disparities if they are appropriately used by related stakeholders such as healthcare providers and health information communications. Also, these literacy profiles can be integrated into the abovementioned AI-augmented lifelong cyberlearning systems and AI-assisted language translation, simplification and summarization systems for self-learning and self-management.

### Conclusion

In response to the COVID-19 pandemic and infodemic, we have discussed major concerns in eHealth literacy education, summarized the significances and challenges of improving eHealth literacy in both communicable (e.g., COVID-19) and non-communicable diseases (e.g., cancer, Alzheimer's disease, and CVDs), and made our recommendations of a general framework of AI-based approaches to improving eHealth literacy and combating infodemic, including AI-augmented lifelong learning, AI-assisted translation, simplification, and summarization, and AI-based content filtering. Finally, we discussed additional key issues including integrating eHealth literacy education into curriculum, a whole-society approach to improving eHealth literacy and combating infodemic, and healthcare disparity. We envision that there are huge opportunities in the near future to both improve eHealth literacy and combat infodemic because of the fast advancements of AI technologies and their wide and rapid adoption in every corner of our society.

## Author Contributions

TL and XX conceptualized this work and prepared the manuscript draft. Both authors contributed to the article and approved the submitted version.

## Conflict of Interest

The authors declare that the research was conducted in the absence of any commercial or financial relationships that could be construed as a potential conflict of interest.

## Publisher's Note

All claims expressed in this article are solely those of the authors and do not necessarily represent those of their affiliated organizations, or those of the publisher, the editors and the reviewers. Any product that may be evaluated in this article, or claim that may be made by its manufacturer, is not guaranteed or endorsed by the publisher.

## Footnotes

^1^ https://en.wikipedia.org/wiki/Health_literacy

^2^ https://www.nsf.gov/funding/pgm_summ.jsp?pims_id=504984&org=CISE

^3^ https://circlcenter.org/resources/reflections-report/ai/

^4^ https://en.wikipedia.org/wiki/Mashup_(education)

^5^ https://health.gov/our-work/health-literacy/national-action-plan-improve-health-literacy

^6^ https://en.wikipedia.org/wiki/GPT-2

^7^ https://www.hon.ch/en/

^8^ https://en.wikipedia.org/wiki/Health_On_the_Net_Foundation

^9^ https://www.cdc.gov/healthliteracy/education-support/schools.html

^10^ http://digitalliteracy.us/
